# Students teaching students: A survey of a medical student led surgical skills workshop - A prospective cohort study

**DOI:** 10.1016/j.amsu.2020.05.034

**Published:** 2020-05-30

**Authors:** Swapnil D. Kachare, Christina Kapsalis, Angelica Yun, Milind D. Kachare, Jared Davis, Dexter Weeks, Joyce Jhang, Bradon J. Wilhelmi, Morton L. Kasdan

**Affiliations:** a Division of Plastic and Reconstructive Surgery, Department of Surgery, University of Louisville, Louisville, KY, United States; b Division of Plastic and Reconstructive Surgery, Department of Surgery, University of North Carolina, Chapel Hill, NC, United States; c University of Louisville School of Medicine, Louisville, KY, United States; d Department of Surgery, Robert Wood Johnson Medical School, New Brunswick, NJ, Canada; e Division of Plastic and Reconstructive Surgery, Department of Surgery, The University of Mississippi, Jackson, MS, United States; f Division of Plastic and Reconstructive Surgery, Department of Surgery, The University of Texas Medical Branch, Galveston, TX, United States; g Department of Surgery, East Carolina University, Greenville, NC, United States

**Keywords:** Skills course, Medical student, Suturing, Knot tying, Mentorship

## Abstract

**Background:**

Surgical skills training is a recognized vital component of medical education, yet a standardized curriculum does not exist. Early opportunities for skills development and mentorship may increase student interest in pursuing surgery. We evaluated the effects of a student-led, faculty-supervised suture clinic on student comfort level with basic surgical skills and interest in surgery.

**Methods:**

A cohort survey study of 103 second-year medical students participating in a surgical skills course was performed between the years 2016–2018. Upon completion of the course, we assessed their comfort level with performing six basic skills as well as their interest in pursuing surgery based on pre- and post-course surveys.

**Results:**

Mean age was 25 years and 50.5% were female. Most students (61.2%) had no prior suturing experience. Upon completion of the course, there was a significant improvement (p = < 0.0001) in comfort level for each of the six skills. Most students (81%) reported an increased interest in surgery.

**Conclusions:**

Early implementation of a basic skills workshop can augment student comfort level and promote an interest in surgery. Peer student mentors can effectively lead the workshop and minimize the time commitment needed by surgical faculty. This can serve as a new direction in medical education and an avenue for further studies to analyze the longitudinal effects of the curriculum on career choice and success in surgical residency.

## Highlights

• Learning suture skills early in medical school leads to future operative confidence.
• Early suture skills teaching can significantly increase skill level among junior medical students.
• Suture clinics can provide students opportunities for mentorship and encourage interest in pursuing surgical specialties.
• Medical Students appropriately trained can lead a suture clinic, minimizing demand on faculty support.

## 1 Introduction

Role models, mentors, and early operative exposure are critical in attracting students to a career in surgery [[1], [2], [3], [4], [5]]. Students considering a future in surgery report that one of the greatest factors in their decision was having a role model [2]. In fact, medical students are twice as likely to be interested in surgery if they have a positive surgical role model [5]. Additionally, both faculty-led and peer-assisted surgical skills workshops targeted toward medical students increase their interest in pursuing a surgical career [1,3]. Every medical school graduate who has matched into a surgical training program is expected to have the a baseline skillset to perform simple, subcutaneous, and mattress sutures prior to the start of residency [6]. Developing proficiency in surgical knot tying and suturing as a medical student, produces interns who display improved confidence and technical aptitude, which can translate into potentially better patient outcomes [7].

It is estimated that 50% of medical schools are not meeting the goals of teaching and evaluating technical procedures [8]. With significant variability in structure and administration of surgical educational curriculums across the United States (U.S.) [9], most medical students lack a formal introduction to surgical skills and techniques. Additionally, immense variability exists in procedural experience amongst medical students [10]. In two separate studies, more than 75% of medical students reported inadequate basic surgical skills teaching during medical school [3,11]. Emphasis on basic surgical and procedural training is often overshadowed by the preclinical curriculum, which is heavily focused on generalized skills and knowledge, such as taking thorough histories and performing appropriate physical exams [11,12]. Furthermore, when surgical skills training is limited to one session at the medical school level, the instructional method often does not lead to consistent, long-term proficiency [13]. Failure to acquire and maintain these necessary technical and procedural skills results in underprepared medical school graduates [14]. According to a survey study, the average first year surgery resident is underprepared to competently and safely perform basic technical skills such as suturing [15]. In a recent study from Nigeria, a medical school implemented a standardized, basic surgical skills program taught by first and second year surgical residents into their medical school education curriculum during their clinical rotations. Upon completion of the curriculum, 98.3% of participants felt very confident in handling basic instruments and 56.7% felt confident in basic suturing proficiency [16].

At the University of Louisville School of Medicine, we have a peer-led suture clinic dedicated to teaching basic surgical skills appropriate for medical students [17]. In addition to developing technical proficiency and comfort with basic surgical skills, another important objective of the suture clinic is to foster students’ interest in surgical careers through peer-teaching and mentorship. The purpose of our study was to evaluate the efficacy of our suture clinic, with respect to student comfort level with technical skills and their interest in surgery. We hypothesized that our surgical skills curriculum would improve student comfort and technical proficiency, as well as, increase interest in surgical fields through early exposure and peer mentorship.

## 2 Materials and methods

The skills curriculum was developed by the senior author, MLK [17]. The course has been ongoing for over 21 years, with over 1000 participants. The supplies and the workspace were provided by the senior surgeon. Students used custom suture boards [18], scissors, needle drivers, forceps, and various sutures. A total of 162 students participated in the course over the study period, from April 2016 to April 2018.

The study has been registered in accordance with the declaration of Helsinki. The Research Registry Unique Identifying Number is researchregistry5551. The research study has also been approved by institutional review board (IRB) at the University of Louisville (IRB # 16.0811) on January 25, 2018. Our work is fully compliant with the STROCSS criteria [19].

### 2.1 Course description

The first session was dedicated to surgical knot tying, focusing on a left two-handed technique and a right one-handed technique. A portion of this session was devoted to teaching students how to properly gown and glove in a sterile fashion, as well as, proper operating room etiquette by volunteer operating room (OR) nurses and technicians (Table 1). Each subsequent session began with 10 min of knot tying practice [17].

**Table 1:** Course description for each of the 4 weekly sessions [17].

Class Session	Skills Taught
1st session	Left two-handed knot Right one-handed knot Gown and gloving
2nd session	Introduction to suture materials Instrument tie Acland video [[18], [20]] Simple interrupted suture Simple continuous suture
3rd session	Acland video [[18], [20]] Buried dermal suture
4th session	Vertical mattress suture Horizontal mattress suture Running subcuticular suture

The second session began with a demonstrative video [20], showing the techniques for proper suturing, followed by a PowerPoint presentation given by a local Board Certified Plastic Surgeon on the different types of suture materials, including their properties and uses. During this second session, students learned simple interrupted and simple continuous running sutures, in addition to proper handling of basic surgical instruments. The third session began with a demonstrational video on how to complete an interrupted deep dermal stitch. Students spent the third session acquiring proficiency in the skills that had were taught in previous sessions [17].

The final session began with a discussion panel held by various surgeons, fellows, residents, and upper level medical students on various topics such as residency, the match process, away rotations, mental health, and well-being. This information session was an opportunity for students to receive mentorship and guidance. The final session also focused on learning vertical and horizontal mattress stitches, and the running subcuticular stitch. Senior surgeons, surgical fellows and residents, OR nurses, surgical technicians, and upper level medical students attended, taught, and provided mentorship and clinical practical information for students during all the sessions [17].

The series of instructional videos on basic surgical suturing skills were created for students by two of the senior faculty, Dr. Morton Kasdan and Dr. Robert Acland [20]. The videos include demonstrations of appropriate needle placement, proper tissue eversion, and symmetric bites of tissue at the appropriate depth across the wound to produce smooth contouring following closure. These videos provided an audio-visual medium to demonstrate proper technique and comprehend the significant concepts of proper tissue eversion and apposition in wound healing [20]. Board Certified Surgeons, surgical fellows, and residents were available to augment the knowledge and technique shown in these videos.

### 2.2 Student selection

Suture clinic instructors were rising 2nd year students selected by the previous year's instructors, who would be entering their 3rd year of medical school. The selection process for instructors involved an application essay, followed by an interview with the previous year's instructors. Primary selection factors included interest in a surgical career, perceived capability to function within a team, and availability during the training and educational sessions. Each clinic had 6-8 students who committed to a 4-week session with classes being held weekly for 2 h. Participants were chosen through a randomized lottery system that was available only to second year students. The cohort selection was performed by a random number generator in Microsoft Excel. The six students with the highest assigned numbers attended the first four sessions of the suture clinic, the next six attended the subsequent four sessions, etc., until the year's schedule was filled. After completing the four sessions, students were given the opportunity to return and practice skills as needed prior to their third- and fourth-year rotations [17].

### 2.3 Survey

The study comprised of students between the years 2016–2018. Participants opted-in to complete an entrance survey, completed prior to beginning the first session, and an exit survey, completed after the final session. The survey was provided to all students in a prospective manner and all the students who participated in the survey study comprised the study cohort. The entrance survey asked students about their motivation for participating in the suture clinic, career goal, experience with previous suturing, participation in an operation, whether they had a surgical mentor, and their goals for the course. Using a 10 point Likert scale, students were also asked to give their comfort level with each of the following skills: (1) using forceps, (2) using needle drivers, (3) one-handed knot tying, (4) two-handed knot tying, (5) instrument tying, and (6) suturing. On the exit survey, students were asked if the goals of the suture clinic were met, if they had an increased interest in pursuing surgery, during which of the 4 sessions they achieved a comfort with knot tying, if they practiced at home, and whether they would recommend the course to other students. Using the same Likert scale from the entrance survey, students were asked to rate their comfort level with each of the 6 tasks at the end of the course. The data obtained from both surveys were used to assess student comfort level with performing basic surgical skills and determine if the goals of the course were met (i.e. interest in surgery and mentorship). Students were informed that the survey was being used to track outcomes of peer mentoring and their identity would be kept anonymous. Students were also given the opportunity to participate in mentoring sessions without completing the survey. To be eligible for inclusion in the cohort study, participants had to be second year medical students who attended all four sessions. A total of 162 students comprised the original population that were provided the optional survey. Subjects who did not complete all four sessions and those who chose to opt-out of the survey were excluded from the study (n = 59), with a total of 103 comprising the final sample size. The margin of error (MOE) using our total population (n = 162) and final sample size (n = 103), with a confidence level of 95%, was 5.8%, which provides a suitable MOE for categorical data in a survey study as described by Bartlett and colleagues [21].

In order to obtain quality control, certain measures taken to ensure consistency included teaching the same curriculum for each group and each year. The second relatively consistent factor was the same supervising mentors throughout all the sessions. The instructors only changed at the start of each academic year. The peer instructors underwent the same course sessions as the subjects, but in order to ensure effective teaching, they required additional training and practice. The instructors were taught the basic skills and techniques by the supervising surgeon, and the supervising surgeon remained the same throughout the cohort study.

### 2.4 Data analysis

Descriptive data were reported as means and ranges to summarize continuous variables or frequencies and proportions for categorical data. Student's t-test paired analyses were performed comparing student comfort level with the various technical skills between the entrance and exit surveys. A significance level of 0.05 was set for all hypothesis tests. All data analysis and manipulation were performed using a combination of Microsoft Excel and GraphPad.

## 3 Results

Participants were 2nd year medical students randomly selected using a lottery who then opted-in to complete the pre- and post-course surveys. Participants who did not complete the course and those who chose to opt-out were excluded from the study. A total of 103 students (63.5%) filled out the surveys with an average age of 25 years. Fifty-one (49.5%) students were male. When asked about the reason for course interest, the majority (58%) stated they planned to become a surgeon or were interested in surgery. Most students (61.2%) did not have prior suturing experience, although the majority of students had previously scrubbed into an operative procedure (65%). Sixty-one (59.2%) students did not have an identified surgical mentor prior to participating in the course. For students interested in the suture clinic, the goals of the students were to improve suturing ability (98.1%), develop mentorship (41.6%), and explore surgical interest (64.1%). When asked to give additional reasons for participating in the course, 50 students (48.5%) responded with the following comments: improve technique, knowledge, and skills, prepare for clinical rotations, and engage in an opportunity for networking.

Student comfort level in performing six basic surgical skills was assessed using a 1–10 Likert scale, with 1 indicating no comfort and 10 indicating expert proficiency. On average, students had a significant improvement in their comfort level upon completion of the course (p = <0.0001). For all six skills, the average comfort level prior to the course ranged from 2.4 (for suturing) to 3.0 (for handling forceps). After course completion, the average comfort level ranged from 7.1 (for handling forceps) to 8.0 (for instrument tying) (Table 2).

**Table 2:** Change in student comfort level for various basic surgical skills pre- and post-skills course. Comfort level for each skill was assessed on an entrance and exit survey using a 1–10 Likert scale, with 1 indicating no comfort and 10 indicating expert proficiency, (n = 103).

Skill	Survey	Comfort Level (mean)	SD	95% CI	p-value
Forceps handling	Entrance	3.0	1.9	−4.53 to −3.76	<0.0001
	Exit	7.1	1.4		
Needle driver	Entrance	2.6	1.7	−4.94 to −4.21	<0.0001
	Exit	7.2	1.4		
1-handed knot tying	Entrance	2.4	2.1	−5.68 to −4.79	<0.0001
	Exit	7.7	1.4		
2-handed knot tying	Entrance	2.5	2.1	−5.48 to −4.54	<0.0001
	Exit	7.6	1.4		
Instrument tying	Entrance	2.5	1.9	−5.96 to −5.09	<0.0001
	Exit	8.0	1.4		
Suturing	Entrance	2.4	1.7	−5.15 to −4.40	<0.0001
	Exit	7.2	1.4		

Upon completion of the course, all students felt the course goals were met and that they had achieved increased comfort with basic surgical skills. Most students (93.2%) stated that they practiced outside of the suture clinic. Over the 4-week session, the majority of students (48.5%) felt comfortable with knot-tying by the end of the second week (Table 3). Our data indicates that most students developed an increased interest in surgery, with the 49 students (47.6%) who were previously interested in surgery strengthening their surgical ambitions. One-hundred percent of students would recommend the course to others. Study participants tolerated the course well, and there were no adverse events throughout the cohort study.

**Table 3:** Course session at which students achieved comfort in knot tying, (n = 103).

Session	n (students)	%
1	20	19.4
2	50	48.5
3	22	21.4
4	11	10.7

## 4 Discussion

There is much concern that medical graduates are underprepared for clinical responsibilities due to a decline in the emphasis of technical teaching, as well as, a lack of curriculum standardization for learning basic surgical skills [11,14]. A survey study by Matheson et al. indicated that among 70 items analyzed, suturing was noted to be one of the skills that first year residents were most underprepared in performing [15]. Despite the importance of mentoring in career advancement for medical students [22], there has been an inadequate number of mentoring programs for medical students [23]. In this study, we demonstrated that second year medical students who were provided dedicated procedural training and access to surgical mentors had a significantly increased comfort in performing basic skills and an increased interest in pursuing a surgical career.

It has been proven that procedural performance and independence leads to feelings of competency [12]. There is a significant association between the frequency of task performance and self-assessed competence [24]. In our study, we demonstrated a significant increase in procedural comfort level among medical students in 6 technical tasks at the completion of the course. Prior to the course, student comfort level with suturing was a 2.4/10 at week 1, which increased to a 7.2/10 after week 4. This significant improvement in suturing was similar to findings by Routt and colleagues who compared improvement in suturing proficiency at 30 days in 1st and 2nd year students, between a control group that received teaching on day 1 only and an experimental group who received teaching on days 1, 10, 20, and 30 (similar to our study population). They found a 0% pass rate in the control group (1 day of teaching) versus a 91.7% pass rate for the experimental group (4 days of teaching over 30 days) [13]. Interestingly in our study population, the majority of students (n = 50, 48%) felt comfortable with knot tying after 2 sessions and an additional (n = 22, 21.4%) felt comfortable after 3 sessions. Gershuni and colleagues assessed proficiency in a number of tasks similar to our study, including suturing, two-handed, one-handed, and instrument tying. Their study also demonstrated significant improvement in proficiency and efficiency [25].

Student-organized, faculty-led surgical skills workshops for medical students have been shown to increase students’ interest in surgical careers amongst those who were initially undecided about surgery [1]. A recent survey study by R. J. Karmali et al. showed that upon completing a peer led mentoring program similar to ours, there was a definite increase from 67% to 78% of students interested in pursuing surgery upon course completion. Additionally, 72% of the participants also stated they would select a different surgical elective [26]. This was similar to the findings in our study in which previously undecided students, who were taught by fellow classmates with supervision by senior surgeons, developed a new interest (33%) in pursuing surgery. Among students who were already interested in surgery, 47.6% expressed an increased interest in pursuing a surgical career. In a survey study of a peer-assisted workshop by Preece and colleagues, students also demonstrated an increased desire to pursue a career in surgery. Similar to our findings, those students who had an interest in surgery prior to the workshop enhanced their desire to pursue surgery at the completion of the workshop [3].

Early surgical exposure has proven to increase students’ interest in applying for a surgical residency [1,3,[27], [28], [29]]. Surgical demonstrations by an anatomy course had a positive impact on first-year medical students' perceptions of surgeons and surgery [27]. Smith et al. found an increased interest in hand surgery in a cohort of students who attended a 1-h presentation by a hand surgeon [28]. Likewise, a 1-h session with a panel of surgeons demonstrated a positive impact on first-year medical students' interest in pursuing a surgical career [29]. A student-organized, faculty-led “Surgery Saturday” workshop described by Patel et al. showed an increased interest in 87% of students who were originally undecided about surgery [1]. Similar increases in interest were found in a study that followed a peer-taught surgery workshop held for second- and third-year medical students [3]. Our study supports these studies with an 80% increase in surgical career interest following the suture course.

There are inherent limitations to both single institution and survey studies. Since participation in this course was voluntary in nature, the course may have self-selected students with pre-existing interest in surgery (58% interested in surgery) and varying levels of surgical experience and exposure. Students’ handedness was not assessed as all students were provided right-handed ratcheted instruments and were taught right-handed suturing. Even though this course was taught by students, there was consistent, on-site faculty supervision. In addition, a randomized control trial has shown that medical students are capable of providing surgical skills teaching with the same efficacy as experienced faculty [30]. Another limitation is the subjective nature of the self-reported surgical skills comfort level, making absolute conclusions difficult. Finding an objective measure of skills and student confidence has been a challenge. However, in a recent study published by the Department of Surgery at Southern Illinois University School of Medicine, over a 10-year period, they developed an objective measure of basic surgical skills called the Verification of Proficiency evaluation instrument. This instrument was used as a method to objectively evaluate PGY-1 surgery residents and their proficiency in basic surgical skills. The study showed that this instrument could potentially be used as a standardized baseline of skill levels and potentially as a predictor of future performance during residency [31]. This instrument could be a method of objective measurement of future studies and contains a standardized list of skills expected of medical students preparing for residency.

## 5 Conclusions

In conclusion, a basic skills course provided to medical students early in medical school can significantly improve their comfort level and increase their interest in surgery. This course can be effectively run by peer mentors and minimize a significant time commitment from surgical faculty. Student-led workshops have grown in popularity and could be a topic of further research in medical education curriculums. Further studies assessing this cohort of students through their third- and fourth-year rotations and into their residency may allow for a quantifiable assessment of long-term comfort level with surgical skills.

## Author contribution

Please specify the contribution of each author to the paper, e.g. study design, data collections, data analysis, writing. Others, who have contributed in other ways should be listed as contributors. Angelica Yun, MD: writing. Bradon J. Wilhelmi, MD: writing. Christina Kapsalis, MD: data analysis & writing. Dexter Weeks, MD: study design & writing. Jared Davis, MD: study design & writing. Joyce Jhang: writing. Milind Kachare: writing. Morton Kasdan, MD: studydesign & writing. Swapnil Kachare, MD: data analysis, study design & writing.

## Guarantor

Swapnil Kachare.

## Funding

This research did not receive any specific grant from funding agencies in the public, commercial, or not-for-profit sectors.

## Declaration of competing interestCOI

There are no conflicts of interest to disclose. This research did not receive any specific grants from funding agencies in the public, commercial, or not-for-profit sectors.

## Provenance and peer review

Not commissioned, externally peer reviewed.
